# Comparison of complete nasal packing with and without integrated airways

**DOI:** 10.12669/pjms.39.5.7478

**Published:** 2023

**Authors:** Afifa Jamil, Hira Abdul Hameed, Atif Hafeez Sidiqqui, M. Shuja Farrukh

**Affiliations:** 1 Afifa Jamil, MBBS. ENT Unit-1, Dr Ruth K.M. PFAU, Civil Hospital, DUHS, Karachi, Pakistan; 2 Hira Abdul Hameed, DLO, RMO. ENT Unit-1, Dr Ruth K.M. PFAU, Civil Hospital, DUHS, Karachi, Pakistan; 3 Atif Hafeez Sidiqqui, FCPS. ENT Unit-1, Dr Ruth K.M. PFAU, Civil Hospital, DUHS, Karachi, Pakistan; 4 M. Shuja Farrukh, FCPS. ENT Unit-1, Dr Ruth K.M. PFAU, Civil Hospital, DUHS, Karachi, Pakistan

**Keywords:** Nasal Packing, Nasal Airway, Septoplasty, Oxygen Saturation, Postoperative Pain

## Abstract

**Objective:**

To compare the effects of nasal packing using a Nasopore nasal packing with and without an airway tube on postoperative pain, SpO_2_, nasal obstruction, and difficulty in breathing complaints.

**Methods:**

This comparative study was conducted at Dow University of Health Sciences (DUHS), DMC Civil Hospital Karachi between September 18, 2021, to May 19, 2022. A total of 70 patients who underwent septoplasty for septal deviation and chronic hypertrophic rhinitis were equally divided into two groups. Group-A patients received nasal packing using a Nasopore nasal packing with an airway tube, and Group-B patients received nasal packing using a Nasopore nasal packing without an airway tube. Post-operation Nasal pain sensations were measured using the Wong-Baker Faces Pain Rating Scale at 2 and 12 hours. SpO_2_ was measured at 30 minutes pre-operatively with an O_2_ saturation monitor and 12 hours post-operatively during sleep.

**Results:**

The postoperative pain at two hours and 12 hours was compared between the two groups, and a significant difference was observed. In Group-A, the average SpO_2_ decreased > 4% from baseline in 5.7% patients, and 37% in Group-B. A significant difference was observed in the severity of nasal obstruction and difficulty breathing, P-value < 0.05.

**Conclusion:**

It is concluded that septoplasty followed by applying nasal packing with integrated airway reduces postoperative pain and improves oxygen saturation compared to nasal packing without integrated airways.

## INTRODUCTION

Nasal septum deviation (DNS) is a common issue that can be congenital, developmental, or traumatic. Most cases are asymptomatic and can affect people of any gender and age, but it is more common among men, and symptoms usually appear in adults and adolescents.^1^ The major symptoms of septal deviation are nasal obstruction causing difficulty in breathing.^2^ Other common symptoms include nasal blockage, bleeding, headache, hypoxemia, sinusitis, hypoxemia, cosmetic deformity, and septoplasty.^3^

The most common form of treatment for symptomatic nasal septal deviation (DNS) is septoplasty.^4^ In otolaryngology clinics all around the world, it is a surgical technique frequently carried out. In order to increase nasal airflow, treat DNS symptoms, and repair the nasal septum’s misalignment, septoplasty is performed. Intra-nasal packing is frequently used as a standard procedure after septoplasty to stabilize the nasal septum and manage bleeding during the first healing phase. Nasal packing includes placing materials into the nasal cavity in order to support the surgical site, tamponade any bleeding points, and preserve its integrity.

The potential effects of nasal packing on oxygenation and sleep breathing must be taken into account, though. Normal sleep breathing patterns can already be hampered by nasal obstruction brought on by DNS, and after nasal packing can make matters worse.^5^ Nasal packing can prevent nasal airflow, which can cause pain, mouth breathing, and even compromise the quality of your sleep. Furthermore, oxygen deprivation and obstructive sleep apnea may be affected by the use of conventional nasal packing materials.^6^ Nasal packing’s obstructive nature has the ability to disturb sleep architecture and interfere with normal breathing patterns while a person is trying to fall asleep. Nasal packing can be done with a variety of materials, such as medicated or Vaseline gauze, paraffin meshes, artificial materials, or even glove fingers. The choice of material is influenced by a variety of variables, including the surgeon’s preferences, supply, and patient-specific concerns. Regarding effectiveness, use, patient comfort, and potential consequences, each material has pros and cons.^7^,^8^

It is worth noting that advances in nasal packing techniques have been made to address some of these concerns. For example, the use of absorbable nasal packing materials, such as Nasopore, has gained popularity. These materials provide temporary support and tamponade while gradually resorbing over time, reducing the need for painful removal.

It can produce significant consequences such as nocturnal hypoxemia, obstructive sleep apnea, aspiration, toxic shock syndrome, and pulmonary oedema.^9^ Ventilating nasal packs, on the other hand, offer to allow the patient to breathe via their nose, preventing all nasal blockage issues.^10^ The superiority of airway integrated versus non-airway integrated nasal packaging has been widely debated. The type of packaging adopted by the surgeon is mainly influenced by habit, familial tradition, or departmental supply.^11^,^12^

However, there is less agreement on the efficiency of septoplasty with nasal packing, both with and without an integrated airway. Furthermore, previous research shows that airway integrated nasal packing has inconsistent effects. The motive behind conducting this randomized controlled trial was to analyze the outcome of airway integrated nasal packing in terms of oxygen saturation enhancement compared to nasal packing without integrated airways, as well as subjective complaints like nasal obstruction and nasal pain sensation as it has not been studied in Pakistani population.

## METHOD

In this comparative study (conducted between September 18, 2021, to May 19, 2022), seventy cases who underwent septal deviation and chronic hypertrophic rhinitis were enrolled via the non-probability consecutive sampling technique. The sample size (n=70) was calculated through WHO software “Sample Size Determination in Health Studies” using a 95% confidence level and 5% margin of error. Postoperative at four hours mean pain score (3.5 vs. 2.6) with unit variance in both groups considered for sample size estimation.^13^ The enrolled participants were then randomly divided into two groups. Randomization was performed by computer-generated random sequences of numbers in blocks of variable length.

### Ethical Approval

It was obtained from the IRB of DUHS, DMC Civil Hospital Karachi (IRB-1988/DUHS/Approval/2021; Dated 15^th^ September 2021), and written informed consent was obtained from all participants.

Group-A received nasal packing using a Nasopore nasal packing with an airway tube, and Group-B received a Nasopore nasal packing without an airway tube. Patients who underwent septoplasty for septal deviation and chronic hypertrophic rhinitis were included in the study. At the same time, patients with a history of nasal surgery, co-morbidities of coronary heart disease, arrhythmia, and chronic obstructive lung disease were excluded. All surgeries were undertaken with GA & followed by a bilateral nasal packing using a Nasopore nasal packing with and without an airway tube. Local anesthesia (subcutaneously injected 2% Lidocaine HCl) was used for the surgery.

The patients were pre-medicated with Intravenous glycopyrolate 10 mcg/kg body weight and Intravenous midazolam 50 mcg/Kg body weight. Classical septoplasty was done with killian incision and the warped cartilage and the bony hump or spur of the septal base were removed. A bilateral Hemiturbinectomy was simultaneously performed for the patients with inferior turbinate hypertrophy. Electrocauterization was used to stop the bleeding from the trimmed turbinates. Continous endoseptal suture with 5-0 polyglycolic acid were used from the bottom to the incision line to prevent septal hematoma and to secure the remaining septal cartilage and bone.

Nasal packing was done with airway integrated Nasopore in Group-A and Nasopore without airway integration in Group-B. For patients in Group-A, a silastic airway with an interior diameter of 5 mm was integrated into the soft nasal packing material. The opening of the nasal airway outside the nostril was funnel-shaped at the rostral end to minimize crust clogging. The nasal airway length was extended 5mm at the caudal end outside the nasal packing material. Preventive oral antibiotics (cephalexin monohydrate 250mg, four times a day), oral antihistamines, and decongestants (loratadine 5mg and pseudoephedrine 120 mg, twice a day) were given to each patient 30 minutes preoperatively and 48 hours post-operatively.

The Subjective complaints about nasal obstruction were recorded using the NOSE (Nasal Obstruction Symptom Evaluation) Score. The participants were asked to describe their pain using the Wong-Baker Faces Pain Rating Scale at two & twelve hours. Post operation. SpO2 was measured at 30 minutes pre-operatively with an O2 saturation monitor and 12 hours post-operatively during sleep. The participants were discharged on the 2nd postoperative day, and the nasal pack was removed after 24 hours post-operatively.

For statistical treatment, the data was statistically analyzed using the SPSS version 23.0, and the quantitative variables were expressed as mean + SD, and qualitative variables were presented as frequency and percentages. Independent t-test and Chi-square/Fisher exact test were used to compare quantitative and qualitative variables in the two groups, respectively. A p-value of less than 0.05 was considered statistically significant.

## RESULTS

The male-female ratio in both groups was almost 50:50, and there was no difference in age distribution between the two groups (p>0.05).Table-I.

**Table-I:** Demographic characteristics of the study subjects.

Variables		Group-A	Group-B	P-value
Age; Mean±SD		26.94±6.9	27.9±7.5	0.57
BMI; Mean±SD		26.6±4.6	27.3±4.0	0.49
Gender; n(%)	Male	18(51.4)	19(44.3)	0.80
	Female	17(48.5)	16(45.7)	
Smoking; n(%)	Smoker	05(14.2)	04(11.4)	0.44
	Non-smoker	30(86.7)	31(88.5)	

Group-A: Nasal packing was done with airway integrated Nasopore; Group-B: Nasopore without airway integration. *p<0.05 is considered significant.

The postoperative pain at two hours and 12 hours was compared between the two groups, and a significant difference was observed as the p-value was < 0.05 (Table-II).

**Table-II:** Comparison of Postoperative Pain Score between two Groups.

Pain Score	Group-A	Group-B	P-value
	Mean±SD	
Postoperative pain at 2 hours	6.0±1.7	9.0±1.0	0.000*
Postoperative pain at 24 hours	3.7±2.1	7.3±0.9	0.000*

Group-A: Nasal packing was done with airway integrated Nasopore;

Group-B: Nasopore without airway integration.

* p<0.05 is considered significant.

In Group-A, the average SpO_2_ decreased > 4% from baseline in 2 (5.7%) patients, and 13 (37%) in Group-B (Table-III).

**Table-III:** Comparison of SpO_2_ between two groups.

Variables	Group-A	Group-B	P-value
	Mean±SD	
SpO_2_ before 30 minutes of surgery	99±0.96	96.0±1.04	0.000*
SpO_2_ after 12 hours of surgery	96.4±1.04	95.4±0.81	0.000*
Reduction in SpO_2_	2.89±0.5	3.2±0.8	0.000*
Reduction > 4%; n (%)	2(5.7)	13(37)	0.000*

Group-A: Nasal packing was done with airway integrated Nasopore;

Group-B: Nasopore without airway integration.

* p<0.05 is considered significant.

When the severity of nasal obstruction and difficulty in breathing were compared, a significant difference was observed, P-value < 0.05 (Table-IV).

**Table-IV:** Comparison of subjective complaints between two groups.

Complains		Group-A	Group-B	P-value
		N(%)	
Nasal Obstruction	Mild	4(11.4)	-	0.000*
	Moderate	29(82.8)	21(60)	
	Severe	2(5.7)	14(40)	
Difficulty in Breathing	None	-	-	0.010*
	Medium	27(77.14)	17 (48.6)	
	Severe	8 (22.8)	18 (51.4)	

Group-A: Nasal packing was done with airway integrated Nasopore;

Group-B: Nasopore without airway integration.

* p<0.05 is considered significant.

## DISCUSSION

This study aimed to compare the outcomes of septoplasty with nasal packing with and without an integrated airway. Nasal packing is commonly used in ENT surgeries to control bleeding and stabilize the nasal structure. However, there is limited evidence and conflicting findings regarding the necessity and benefits of nasal packing after septoplasty^14^-^19^. The study found that patients who underwent septoplasty with nasal packing without an integrated airway reported lower pain scores at the 2nd and 12th-hour postoperatively compared to those who received nasal packing with an integrated airway. These results contribute to the existing knowledge on the topic and suggest that nasal packing without an integrated airway may be a viable option for managing patients undergoing septoplasty. Interestingly, Yu et al., in their study revealed notable differences in postoperative pain reduction between the two groups.^15^ Patients in Group-2, who received Nasopore packing without an integrated airway, reported lower pain scores compared to Group-1 patients, who received Nasopore packing with an integrated airway. This discrepancy suggests that the presence of the integrated airway may have compromised the softness of the packing material. Furthermore, a related study that found that Group-A (with packing) experienced more pain than Group-B (without packing) did further corroborated these conclusions.^20^

There was a significant difference in the severity of nasal obstruction and difficulty breathing among the Group A and B patients. Similarly, another study reported that almost all patients in Group-A (nasal packing without airway integration) had nasal obstruction with dry mouth, difficulty swallowing and disturbed sleep.^16^ Only 33-40% of patients in Groups B (nasal packing with integrated airway) had similar complaints.^16^

In the present study, the average SpO_2_ decreased > 4% from baseline in 2 (5.7%) patients of group-A, and in 13 (37%) patients of Group-B. Similar results were found in another study, the average SpO_2_ (arterial oxyhemoglobin saturation when measured using pulse oximetry) levels were significantly reduced in 33% of the patients that slept with nasal packing without an integrated airway but were within normal limits for all patients with airway integrated nasal packing.^16^ Conversely, the other study^15^ found that SpO_2_ was not significantly different in patients who have undergone septoplasty with nasal packing with and without airway integration.

These differences might have pertained to the different time measurements of SpO_2_. They monitored SpO_2_ during sleep, particularly overnight, and some researchers checked the blood gas before and after the application of nasal packing. Few studies assessed SpO_2_ on the 4^th^, 6^th^, 12^th^, 18^th^, and 48^th^ post-operatively. In the present study, we assessed 12 hours post-operatively during sleep, supporting the attenuation of arterial hypoxia by using an integrated airway with nasal packing materials.

It was not difficult for patients with long-term nasal obstructions to adapt to breathing primarily through the mouth after surgery.^21^ Although the reduction in SpO_2_ might be statistically significant, this amount of reduction in SpO_2_ was clinically irrelevant for the patients in both groups. The ease of adapting to breathing primarily through the mouth after surgery may contribute to a lack of clinically relevant SpO_2_ differences between the two groups.

Overall, by providing a comparative assessment of nasal packing methods, grading postoperative discomfort, tracking oxygen saturation levels, and evaluating nasal blockage and breathing difficulty, this study contributes new knowledge to the existing local literature. These findings contribute to the creation of evidence-based postoperative care strategies and deepen our understanding of the management of patients having septoplasty.

### Limitations

Single-center and a small sample were among the major limitations of the study. Secondly, non-probability consecutive sampling technique was used, and this may not allow generalization of results to the population, and thirdly, we excluded patients with cardiopulmonary disorders that could lead to myocardial infarction or a stroke. As a result, in patients with cardiovascular or pulmonary illnesses, hypoxemia caused by upper airway blockage may be severe enough to produce catastrophic problems.

## CONCLUSION

Septoplasty followed by application of nasal packing with integrated airway reduces postoperative pain and complications and improves the oxygen saturation as compared to nasal packing without integrated airways. Thus, septoplasty with airway integrated nasal packing is much safer and comfortable procedure for patients, so it should be preferred by the surgeons.

### Authors Contribution:

**AF, HAH, AHS** & **MSF:** Are responsible for the concept and study design. **AHS:** Contributed to the data collection and literature review. **AF, HAH, AHS** & **MSF:** Are responsible for data analysis and interpretation and drafting of the manuscript. **MSF, AJ** & **HAH:** Contributed to the critical review, revision and final approval of the study. All the authors are equally responsible and accountable for the accuracy and integrity of the work.
